# The crosstalk between autophagic and endo-/exosomal pathways in antigen processing for MHC presentation in anticancer T cell immune responses

**DOI:** 10.1186/s13045-017-0534-8

**Published:** 2017-10-23

**Authors:** Liangshun You, Liping Mao, Juying Wei, Shenhe Jin, Chunmei Yang, Hui Liu, Li Zhu, Wenbin Qian

**Affiliations:** 1 Department of Hematology, The First Affiliated Hospital, College of Medicine, Zhejiang University, Hangzhou, Zhejiang 310003 China; 2 Institute of Hematology, The First Affiliated Hospital, College of Medicine, Zhejiang University, 79# Qingchun Road, Hangzhou, 310003 Zhejiang People’s Republic of China; 3 Malignant Lymphoma Diagnosis and Therapy Center, The First Affiliated Hospital, College of Medicine, Zhejiang University, Hangzhou, Zhejiang 310003 China

**Keywords:** Autophagy, Endosome, Exosomes, Immune, MHC presentation, Cancer, Immunotherapeutic

## Abstract

T cells recognize antigen fragments from proteolytic products that are presented to them in the form of peptides on major histocompatibility complex (MHC) molecules, which is crucial for the T cell to identify infected or transformed cells. Autophagy, a process that delivers cytoplasmic constituents for lysosomal degradation, has been observed to provide a substantial source of intra- and extracellular antigens for MHC presentation to T cells, which will impact the tumor-specific immune response. Meanwhile, extracellular components are transported to cytoplasm for the degradation/secretion process by the endo-/exosomal pathway and are thus involved in multiple physiological and pathological processes, including immune responses. Autophagy and endo-/exosomal pathways are intertwined in a highly intricate manner and both are closely involved in antigen processing for MHC presentation; thus, we propose that they may coordinate in antigen processing and presentation in anticancer T cell immune responses. In this article, we discuss the molecular and functional crosstalk between autophagy and endo-/exosomal pathways and their contributions to antigen processing for MHC presentation in anticancer T cell immune responses.

## Background

In eukaryotic cells, MHC presentation monitors two proteolytic routes: the ubiquitin-proteasome and the lysosomal systems. Both of these systems are involved in the degradation of endogenous and exogenous antigens. The lysosomal system degrades and recycles long-lived proteins and defective organelles [1, 2], in which extracellular components and plasma membrane receptors are transported to the degradation/secretion pathway by the endo-/exosomal pathway, whereas intracellular components are transported to the lysosome by the autophagy process [3, 4]. Autophagy and endo-/exosomal processes differ mainly on the molecular pathway by which the products (cargo) are delivered to lysosomes for degradation but closely interact with each other at multiple key checkpoints [5].

Macroautophagy (hereafter referred to as autophagy), a cellular self-consumption process, is the main form of autophagy. Basal autophagy enables cells to recycle cytoplasmic constituents and restore metabolic homeostasis, thereby maintaining cellular survival [6]. Aberrant regulation of autophagy has been implicated in the pathogenesis of diverse disease states, such as neurodegenerative disorders [7], microbial infection [8], endocrine diseases [9], myopathies [10], cardiovascular diseases [11], aging [12], and cancer [13]. Except for its basal function, autophagy is readily induced in harsh conditions, including nutrient deprivation, radiation, metabolic stress, endoplasmic reticulum (ER) stress, and chemotherapeutic agents [14]. The role of autophagy as an alternate energy source, and thus as a cell survival mechanism under stressful conditions, is well recognized. Accumulating evidence has revealed that the autophagy pathway and its interacting proteins substantially impact several aspects of innate and adaptive immunity [15, 16]. The immune system uses autophagy to detect invading pathogens and monitor transformed cells. The specific roles of autophagy in innate immunity, which is regulated by pattern recognition receptor (PRR) signaling, are regulating inflammation and eliminating apoptotic corpses to prevent insufficient inflammatory or excessive inflammatory responses [15, 17]. In adaptive immunity, the autophagy pathway is essential to antigen presentation, thymus selection, lymphocyte development, and immune homeostasis [18, 19].

Autophagy has also been implicated in the exosome secretory pathway [20]. An exosome is a kind of small nanometric membrane vesicle that is released to the extracellular environment by almost every cell type. As important mediators in intercellular communications, exosomes manage the exchange of proteins and genetic material derived from parent cells. Evidence shows that this kind of intercellular communication by exosomes is involved in multiple physiological and pathological processes, including immune responses [21–23]. In particular, the communications between immune cells and cancer cells via exosomes play dual roles in modulating tumor immunity [21].

Recent studies suggest that autophagy and endo-/exosomal pathways are closely involved in antigen processing for MHC presentation, which results in the activation of tumor-specific T cells. However, thoroughly understanding the inter-regulations between autophagy and endo-/exosomal pathways in antigen processing is an interesting challenge. In this review, we focus on the crosstalk between autophagy and endo-/exosomal pathways and their contributions to antigen processing for MHC presentation in cancer.

## Overview of autophagy

**Fig. 1:**
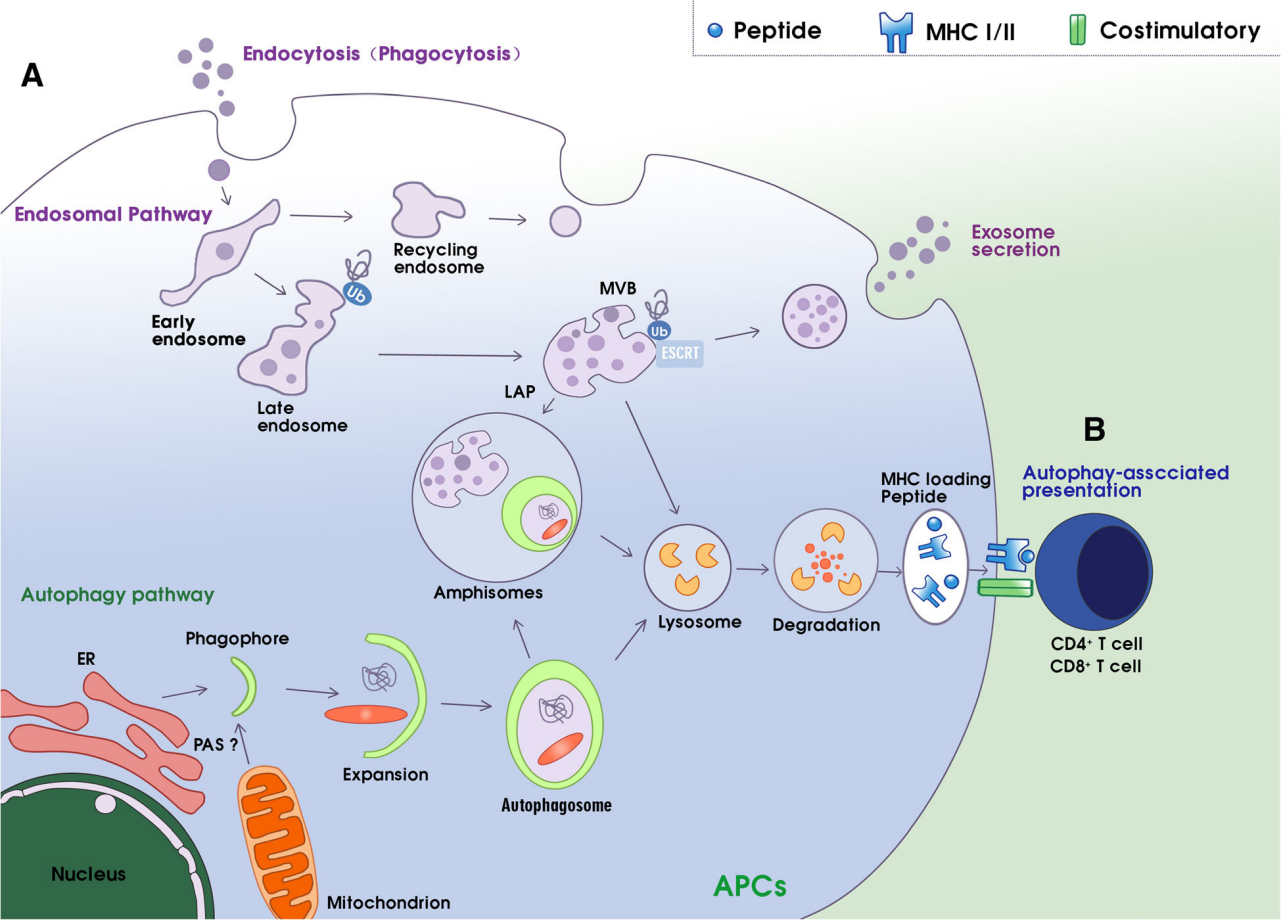
Schematic diagram of the interplay between the autophagy-lysosomal and endo-/exosomal pathways and autophagy-associated antigen presentation. (**A**) The selective degradation of damaged or toxic material, including proteins, by the autophagy-lysosomal or endo-/exosomal pathways are coordinated processes that participate in protein homeostasis and contribute to antigen processing for MHC presentation. The two pathways converge with many common components, especially those that are involved in amphisome formation and the LAP process. Autophagy can regulate endosomal secretion to form extracellular vesicles, which can also regulate autophagy in a paracrine manner. (**B**) Autophagy is a novel pathway for endogenous and exogenous antigen presentation. Autophagosomes recruit cytosolic antigens to endosomal MHC loading compartments via lysosomal degradation and then present peptide-MHC to CD4^+^ or CD8^+^ T cells with the assistance of the costimulatory molecules. Abbreviations: APCs antigen presenting cells, ER endoplasmic reticulum, MHC major histocompatibility complex, LAP LC3-associated phagocytosis, MVB multivesicular bodies, PAS phagophore assembly site

More than 30 autophagy-related gene (ATG) proteins are involved in the complex processes of autophagosome formation, encapsulation of target cargoes, and subsequent fusion with the lysosome for degradation [24, 25]. Autophagosome formation is a multistep process involving at least three stages [18, 25]: initiation, nucleation, and expansion of the isolation membrane (Fig. 1 (A)). The initiation begins with the formation of the phagophore assembly site (PAS), the origin of which is still unclear in mammals [26]. The UNC51-like kinase (ULK) complex, consisting of ULK1 (or ULK2), ATG13, ATG101, and focal adhesion kinase family interacting protein of 200-kDa (FIP200), creates the PAS [27]. When cells are stimulated by autophagy, type I PI3K-AKT-mTOR signaling is inhibited and type III PI3K mammalian vps34/Beclin1 (ATG6) is activated. Inhibition of mTOR re-associates dephosphorylated ATG13 with Atg1, which induces redistribution of mAtg9 from trans-Golgi to late endosome [28]. Simultaneously, the activation of vps34/Beclin1 generates phosphatidylinositol 3-phosphate (PIP3) on the endomembrane, resulting in the isolation and binding of ATG5 and ATG16 to a small template membrane, which is designated as the phagophore [29, 30]. Subsequent nucleation and recruitment of ATG5-ATG12-ATG16L to the autophagosome membrane facilitates the conjugation of phosphatidylethanolamine (PE) to microtubule-associated protein 1 light chain 3 (MAP-LC3) [30–33]. PE-conjugated MAP-LC3 is required for expansion of autophagosome membranes, recognition of target cargo, and fusion of the autophagosome with lysosomes [18]. The autophagosome then fuses with endocytic and lysosomal compartments, ultimately leading to formation of the autolysosome, where engulfed components are eventually degraded [18].

## The crosstalk between autophagy and exosomal pathways

Perpendicular to the autophagy process is the endosomal pathway. Numerous studies have shown a close relationship between the autophagy pathway and the biogenesis and secretion of exosomes [5, 20, 34, 35]. Autophagosomes must undergo a series of maturation steps, in part by fusing with endocytic vesicles, including early and late endosomes and multivesicular bodies (MVBs) [26, 36, 37]. Proper maturation of the autophagosome requires an intact endocytic trafficking pathway, components of the endosomal sorting complex required for transport (ESCRT) pathway, and components involved in endocytic vesicle fusion [38, 39]. ESCRT mutants failed to complete autophagic maturation due to the lack of autophagosome fusion with the endolysosomal system and resulted in an increased number of autophagosomes [40, 41]. Remarkably, autophagy modulators regulate MVB formation and exosome release (Fig. 1 (A)). MVBs are derived from endosomes by inward budding of their membrane to create intraluminal vesicles (ILVs) [42]. Once formed, MVBs can go through the secretory or lysosomal pathway. In the secretory pathway, the MVB can fuse with the plasma membrane to release its intraluminal vesicles as exosomes directly into the extracellular space. In the lysosomal pathway, the MVBs fuse with a lysosome, or alternatively with the autophagosome, to become an amphisome prior to fusion with a lysosome, ultimately leading to content degradation [35, 43, 44]. In autophagy induction, MVBs are directed to the autophagic pathway with consequently greater autophagic degradation and inhibition of exosome release. Alternatively, in a blockage of autophagosome maturation or fusion with a lysosome, the equilibrium would be shifted toward the endo-/exosomal pathway through the fusion of autophagosomes to MVBs and release in exosomes. This dynamic interaction between these interconnected pathways may be of great significance in the context of cellular stress [43, 45].

Phagocytosis (Fig. 1 (A)), a prominent endocytic pathway, is regulated by ATG proteins [46]. During this LC3-associated phagocytosis (LAP) process, MAP-LC3 seemed to be transiently recruited to a subset of the phagosome membrane, which is surrounded by pathogen-associated molecular pattern (PAMP) receptors, thus enhancing phagosome fusion with lysosomes [47, 48]. The generation of reactive oxygen species (ROS) produced by NADPH oxidases-2 (NOX-2) at the phagosome was proposed to be necessary in maintaining the conjugation of MAP-LC3 to phagosomes in LAP [48]. The fate of these phagosomes depends on their cellular background. In plasmacytoid dendritic cells (pDCs) and human macrophages, LAP vesicles seem to be stabilized for fusion with toll-like receptors (TLRs) that contain endosomes and postponed the presentation of extracellular antigens for MHC class II [49, 50]. Thus, the autophagy machinery that mediates LAP can affect the fate of phagosomes and the processing of exogenous antigens.

## Autophagy and antigen presentation in cancer

Recent accumulating evidence has shown that the autophagy pathway plays a crucial role in antigen processing (Fig. 1 (B)). Cancer cells use autophagosome formation to fuse endogenous and exogenous antigen processes with MHC I and II for antigen presentation to T cells, which is of great significance in antitumor immune response [18, 51].

Autophagy can deliver cytoplasmic constituents for lysosomal hydrolysis, which contributes to the processing of endogenous antigens for presentation by MHC II molecules [52, 53]. Some previous studies revealed that antigens, including tumor antigens, can be presented on MHC II molecules. For example, the agents specifically blocking autophagy (3-MA and Wortmannin) were shown to reduce the capacity of dendritic cells (DCs) to present MHC II-restricted peptide derived from endogenously synthesized mucin1 (MUC1), which is a heterodimeric protein that is aberrantly expressed in various cancer cells [54–56]. It is likely that some anticancer drugs potentially act by triggering autophagy and, by doing so, could cause an enhanced presentation of intracellular CD4^+^ T cell epitopes in MHC II-expressing tumor cells. These studies demonstrated that autophagy facilitates MHC II presentation of peptides from intracellular proteins in a general way and indicated that autophagy might act as a potential mechanism for the presentation of tumor antigen to MHC molecules.

Different from antigen processing for MHC II presentation, the role of autophagy in antigen processing for MHC I presentation is not well studied. However, autophagy machinery has been implicated in the presentation of exogenous, endocytosed antigens by MHC class I molecules and is a pathway termed cross-presentation that plays a critical role in cytotoxic T cell immunity against tumors. Several studies reported the relationship between MHC I-mediated autophagy and cancer immune response. The direct evidence from Li et al. [57] showed that in HEK 293 T cells expressing ovalbumin (OVA) antigen treated with mTOR, inhibitor rapamycin underwent autophagy and displayed elevation of the MHC class I cross-presentation of OVA antigens by DCs. A recent study discovered that TNF-α could induce autophagy to enable the processing and presentation of mitochondrial antigens at the cell surface by MHC class I molecules [58]. Collectively, autophagy has been suggested to contribute to the cross-presentation of MHC I molecules, which plays a pivotal role in the initiation and development of T cell immune responses to tumor-associated antigens, including self or mutated self-antigens derived from tumor cells.

## Exosome-mediated activation of immune response via antigen presentation to T cells

Exosomes are a kind of nanometric (30–120 nm in diameter) extracellular vesicles (EVs) formed in vesicular bodies in the endosomal network and can be released by almost all types of cells, including cancer cells. Exosomes play an essential role in cell-to-cell communication, both locally and systemically, by exchanging of their contents, including a subset of proteins, lipids, and functional genetic material derived from the parent cells [59–61]. Emerging evidence shows that intercellular communication mediated by exosomes is involved in pathological processes of many diseases, especially in cancers. Interestingly, exosomes have been observed to play crucial roles in carrying and presenting functional MHC-peptide complexes to modulate antigen-specific T cell activation through direct presentation and cross-presentation pathways [21, 62–64]. In this section, we focus on exosome-mediated activation of anticancer immune response via MHC presentation to T cells.

### Dendritic cell-derived exosome (DEX)-mediated antigen presentation

Through intercellular communication, exosomes stimulate the immune system to produce antitumor responses, of which the key factor is the APCs, which present MHC-peptide complexes to T cells. Initial studies of the proteome of DEXs revealed a unique molecular composition that endows them with strong immunostimulatory properties in antigen processing and presentation [65]. In 1996, B cell-derived exosomes were first identified as possessing antigen-presentation machinery on their surface membranes and the ability to induce antigen-specific MHC II-restricted T cell immune responses [65]. Subsequently, this phenomenon was discovered to be shared by DEXs, which carry surface MHC class I and MHC class II molecules, and therefore can potentially directly stimulate CD8^+^ and CD4^+^ T cells against cancer cells, respectively [66]. Furthermore, DEXs derived from tumor peptide-stimulated DCs could be used to prime tumor-specific cytotoxic T lymphocyte (CTL) responses that could control, or in some cases eradicate, established murine tumors [65]. Additionally, DEXs were shown to possess some kind of functional molecular substance on its surface that may participate in antigen presentation. CD86, a functional costimulatory molecule, may contribute further toward aiding T cell priming during antigen presentation [67–69]. Heat shock protein 70 (Hsp70) family members, another DEX component presented in endocytic compartments of DCs, are in charge of a part of immunogenicity, given their antigens’ chaperone and MHC-loading roles [70].

**Fig. 2:**
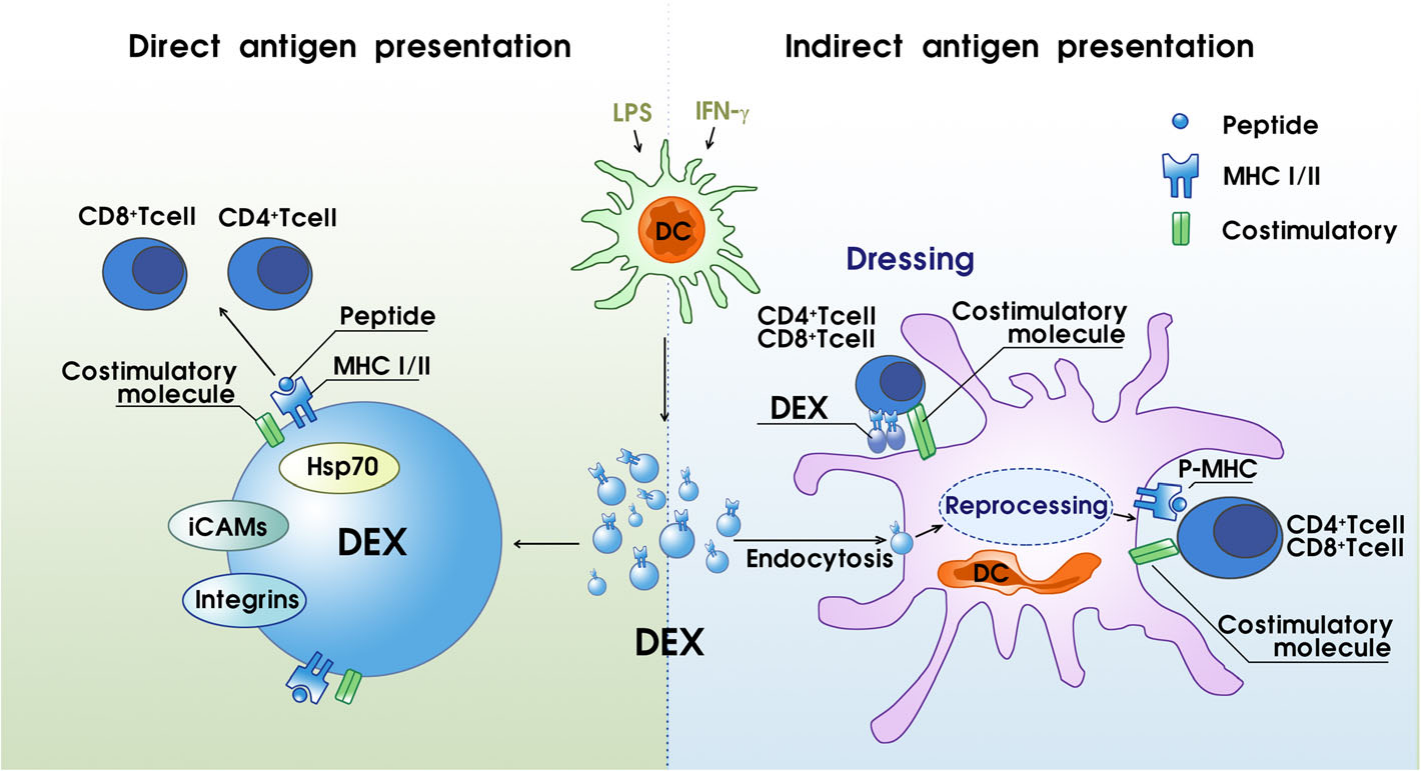
DEXs stimulate T cells via direct and indirect antigen presentation processes. MHC I and MHC II molecules and peptides on the surface of DEXs can be directly presented to T cells, thereby activating T cells. The costimulatory molecules on the surface of DEXs aid this process. Indirect DEX-to-T cell stimulation via bystander DCs is a far more efficient pathway. Two possible mechanisms have been observed in the indirect presentation process. One may be called reprocessing. In this process, the DEX-MHC antigens are captured and reprocessed by APCs and act as the APC-MHC antigens. The other process, known as cross-dressing, is still debated. DEX peptide-MHC complexes attach to mature APC surfaces, which provide the required costimulatory molecules that are absent in the DEXs, and thus can be recognized by T cells directly without the need of APC reprocessing. Abbreviations: DCs dendritic cell, DEXs dendritic cell-derived exosomes, Hsp70 heat shock protein 70, LPS lipopolysaccharide, MHC major histocompatibility complex, pMHC peptide-MHC, iCAMs intercellular cell adhesion molecules

Two mechanisms have been proposed for how DEXs present antigens via their MHC molecules to stimulate T cell responses: direct and indirect pathways (Fig. 2). It was shown that DEXs can directly stimulate T cells in vitro, although it appears that this mechanism operates much more efficiently in stimulating T cell lines, including activated and memory T cells, compared with naive T cells [66, 71–73]. Direct DEX-to-T cell stimulation appears to be more inefficient in priming naive T cells than T cells of the parent APCs, but it can be improved if DEXs are immobilized or their concentration is increased in vitro [74].

Indirect DEX-to-T cell presentation following interactions of DEXs and DCs is another pathway that stimulates T cell responses and is likely to be the most fundamental pathway in vivo (Fig. 2) [63, 71, 75]. Of particular note, DEX priming of naive T cells has been shown to occur only if APCs are present [66, 76]. The presence of certain exosome surface membranes, such as integrins and intercellular cell adhesion molecule-1 (iCAM1, also known as CD54), facilitates the uptake of DEXs by APCs [71, 77]. Indeed, the exosomes released from mature DCs treated with lipopolysaccharide (LPS) or IFN-γ possess more surface expression of MHC class II, CD86, and iCAM1 molecules and exhibit a more potent T cell stimulatory function than exosomes secreted by immature DCs [68, 78–80]. To date, two possible mechanisms have been described for indirect DEX-to-T cell stimulation via bystander DCs. One indirect presentation mechanism, which has been proven and approved, may temporarily be called “reprocessing.” In this process, the DEX-MHC antigens are captured and reprocessed by APCs and act as the APC-MHC antigens [71, 78]. In the other process, known as “cross-dressing,” DEX peptide-MHC complexes attach to mature APC surfaces, which provide the required costimulatory molecules that are absent in the DEXs, and can thus be recognized by T cells directly without the need of APC reprocessing [76, 81]. However, the “cross-dressing” process is still debated and must be further investigated.

### Modified tumor-derived exosome (TEX)-mediated tumor-specific antigen presentation and tumor vaccine

Different from DEXs, in terms of the immune system, TEXs play dual roles in modulating tumor immunity: immunosuppression and immune activation. TEX properties are distinct from the properties of exosomes secreted by normal cells, except TEXs are rich in various immunosuppressive molecules. TEXs also carry tumor-associated antigens (TAAs), a variety of co-stimulatory proteins and MHC molecules, all of which enable them to stimulate immune responses [82, 83]. The “yin and yang” of TEXs in the regulation of tumor immunity are summarized in a review by Liu et al. [21]. In this section, we focus on TEX-mediated tumor-specific antigen presentation in the antitumor immune response.

Early studies showed that TEXs containing native tumor antigens can be efficiently transferred to DCs and induce antigen-specific CD8^+^ T cell activation via the reprocessing or cross-dressing process, which results in tumor rejection in various prophylaxis and therapeutic murine tumor xenograft models [84–87]. Moreover, vaccination of mice with TEXs was shown to induce a potent CD8^+^ T cell-mediated antitumor effect not only on the autologous tumor, but also against other related tumors expressing the same tumor-rejection antigens [77]. Another approach to exploit exosome-based cancer immunotherapy is the application of DCs pulsed with tumor peptides [88–91]. Both mouse and human TAA-loaded DCs can secrete exosomes that express functional MHC class I, class II, and T cell co-stimulatory molecules. These exosomes have been reported to stimulate tumor-specific CD8^+^ T cells in vivo and inhibit tumor growth in mice. On the basis of these clues, TEXs have been developed as cancer-specific vaccines for clinical application. In fact, TEX vaccines from patients with metastatic melanoma, advanced colorectal cancer, and non-small cell lung cancer have been tested in phase I and/or phase II clinical trials [92–97].

**Fig. 3:**
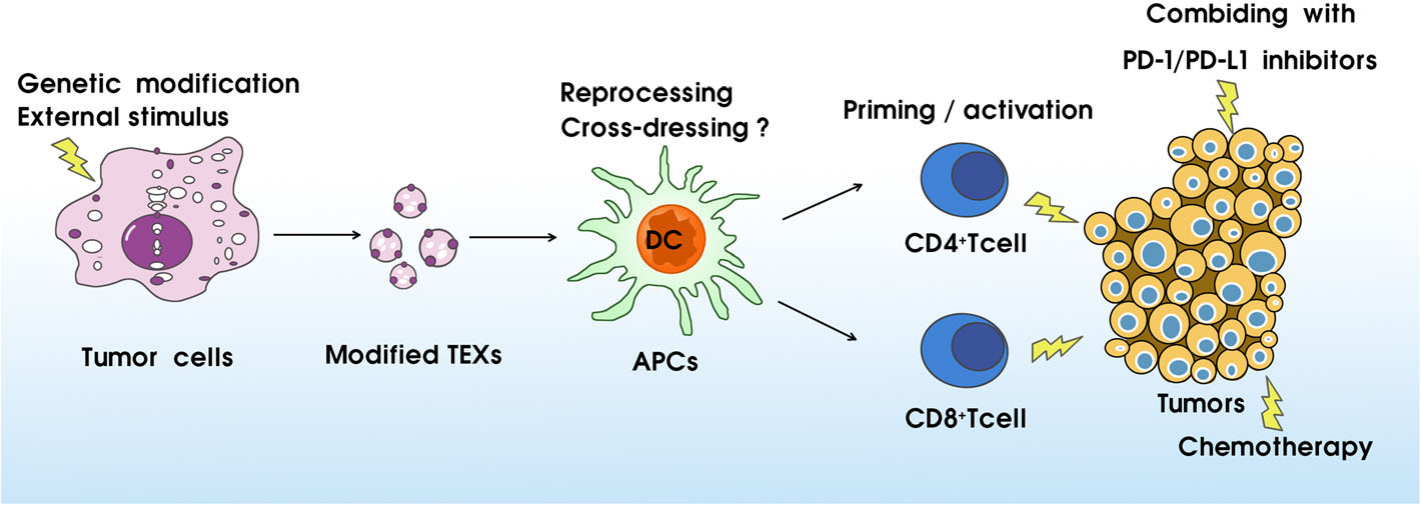
Modified TEX-mediated tumor-specific antigen presentation. To elicit a more efficient antitumor immune response, one strategy is to make genetic modifications to the original cells, such as CD40L- or cytokine gene (IL-2 and IL-18)-modified cancer cells, or add external stimulus to drive tumor cell release of more immunogenicity exosomes. Other strategies involve the combined treatment with TEXs and PD-1/PD-L1 inhibitors or chemotherapy. Abbreviations: APCs antigen presenting cells, TEXs tumor-derived exosomes, PD-1 program death-1, PD-L1, program death ligand-1

However, the antitumor immune responses induced by TEXs are mild, and thus, many strategies have been adopted to develop modified TEXs to elicit a more efficient antitumor immune response (Fig. 3). One of the common strategies is to make genetic modifications to the original cells to improve the immunogenicity of exosomes, such as CD40L- or cytokine gene (IL-2 and IL-18)-modified cancer cells [98, 99]. Other strategies involve adding external stimulus, such as tumor-specific antigens [100–102], to trigger tumor cells to release more effective specific exosomes. Of particular note, combining treatment involving TEXs and program death-1 (PD-1) or program death ligand-1 (PD-L1) blockades could reduce tumor-infiltrating lymphocyte (TIL) suppression and enhance T cell priming [103–105]. Moreover, a recent study showed that TEXs combined with chemotherapy agent cyclophosphamide (CTX) significantly enhanced tumor antigen-induced CD8^+^ T cell recall responses in vivo, leading to a synergistic effect against pre-established tumors [106].

## Conclusions

The notion that autophagy and endo-/exosomal pathways are distinct should be reconsidered because they share many components and are intertwined in a highly intricate manner. In essence, autophagy can regulate endosomal secretion to form extracellular vesicles, which can in turn regulate autophagy in a paracrine manner. Recent studies suggest that autophagy plays such a role in the context of anticancer T cell immune responses, while exosomes have been observed to play crucial roles in carrying and presenting functional MHC-peptide complexes to modulate tumor-specific T cell activation. Therefore, we predict that antitumor immune responses could be regulated by modulating the molecular interactions between the autophagy and endo-/exosomal pathways according to the status of cellular metabolism. Despite the major challenges that may be encountered in further investigation of the precise regulation of these two pathways to achieve the expected effective anticancer immune response, the prospect of autophagy- and exosome-associated immunotherapy as a novel cancer treatment remains highly promising.

## Abbreviations

APCs: Antigen-presenting cells
ATG: Autophagy-related gene
CTL: Cytotoxic T lymphocyte
CTX: Cyclophosphamide
DCs: Dendritic cells
DEXs: Dendritic cell-derived exosomes
ER: Endoplasmic reticulum
ESCRT: Endosomal sorting complex required for transport
EVs: Extracellular vesicles
FIP200: Focal adhesion kinase family interacting protein of 200-kDa
Hsp70: Heat shock protein
iCAM1: Intercellular cell adhesion molecule-1
IL: Interleukin
ILVs: Intraluminal vesicles
LAP: LC3-associated phagocytosis
LPS: Lipopolysaccharide
MAP-LC3: Microtubule-associated protein 1 light chain 3
MHC: Major histocompatibility complex
mTORC1: Mammalian target of rapamycin complex1
MUC1: Mucin1
MVBs: Multivesicular bodies
NADPH: Nicotinamide adenine dinucleotide phosphate
NOX-2: NADPH oxidases-2
PAMP: Pathogen-associated molecular pattern
PAS: Phagophore assembly site
PD-1: Program death-1
pDCs: Plasmacytoid dendritic cells
PD-L1: Program death ligand-1
PE: Phosphatidylethanolamine
PIP3: Phosphatidylinositol 3-phosphate
PRRs: Pattern recognition receptors
ROS: Reactive oxygen species
TAAs: Tumor-associated antigens
TEXs: Tumor-derived exosomes
TIL: Tumor-infiltrating lymphocyte
TLR: Toll-like receptor
ULK: UNC51-like kinase
vps34: Vacuolar protein sorting 34
